# Protective effect of selenium and vitamin C on the fertility of male rats given penconazole

**DOI:** 10.5935/1518-0557.20230042

**Published:** 2024

**Authors:** Elaheh Shams, Vahideh Abdollahi, Mozhgan Harfsheno, Seyedeh Ommolbanin Ghasemian

**Affiliations:** 1 Behbahan Faculty of Medical Sciences, Behbahan, Iran; 2 Department of Animal biology, Kharazmi University, Tehran, Iran; 3 Department of Biology, Faculty of Science, Shahid Chamran University of Ahvaz, Ahvaz, Iran; 4 Department of Veterinary, Behbahan Branch, Islamic Azad University, Behbahan, Iran

**Keywords:** selenium, vitamin C, penconazole, rat testicular tissue

## Abstract

**Objective:**

Penconazole is used in agriculture and human and veterinary medicine applications. It has been included in the acute toxicity hazard category by the WHO. This study examines the protective effect of selenium and vitamin C on the fertility of male rats given penconazole.

**Methods:**

Nine groups of rats were given penconazole at concentrations of 50 and 75 mg/ml and selenium and vitamin C at concentrations of 0.5 and 100 mg/ml, respectively. Serum levels of LH and FSH were measured with ELISA kits; β-actin, GPX4, AQP7, PRM2, and BAX gene expression was evaluated with real-time PCR performed on the left testis of each rat.

**Results:**

LH, FSH, and testosterone levels were lower in the groups given penconazole (50 and 75 mg/kg). Histopathology showed that the groups given penconazole had the lowest number of spermatogonia and primary spermatocytes; these numbers were greater in the groups receiving penconazole together with selenium or vitamin C; and the highest counts were observed in separate groups given Se and vitamin C. GPX4, AQP7, PRM2 and BAX gene expression in the groups receiving penconazole was different from controls and was modulated by treatment with selenium or vitamin C.

**Conclusions:**

This study showed that antioxidant compounds have a strengthening effect on the reproductive system and can mitigate the destructive effects of chemical fungicides.

## INTRODUCTION

Exposure to pesticides may produce negative effects on endocrine glands, mutagenicity, carcinogenicity, and liver, kidney, and central nervous system toxicity (Zhan *et al*., 2020). Triazole fungicides have been associated with a number of reproductive effects in males. Penconazole [1-(2-(2,4-dichlorophenyl)-pentyl)-1H-1,2,4-triazole] is a systemic fungicide from the triazole family, which is used to control the development of fungi on fruits and vegetables and inhibit diffusion by interfering with cell membrane sterol biosynthesis (Icoglu Aksakal & Ciltas, 2018; Chandra & Roychoudhury, 2020). Continuous exposure to penconazole significantly reduces testosterone hormone levels, causes changes in the testicles at the level of the spermatogenic cells, and significantly deteriorates the morphology of Sertoli, Leydig, and spermatogenic cells (Jia *et al*., 2021). Many studies showed the beneficial effects of antioxidants on sperm quality. Selenium is a non-metallic element that plays an essential role in enzyme function and acts as an antioxidant in various tissues. Selenium deficiency decreases sperm parameters such as motility (Zoidis *et al*., 2018). Selenium exists in different chemical forms and is often associated with sulfur compounds (Zoidis *et al*., 2018; Kiełczykowska *et al*., 2018). It is an essential element in testicular function (Saito, 2020; Ramírez-Acosta *et al*., 2022).

The testicle is one of the most important target organs for selenium. Selenium concentrations increase during puberty and with the onset of spermatogenesis (Hamza & Diab, 2020; Santos-Sánchez *et al*., 2019). The testicles contain various antioxidant enzymes for protection against harmful effects. Vitamin C acts as a potent water-soluble antioxidant in body fluids that plays an important role in the inhibition of free oxygen radicals and cell membrane stability (Ramírez-Acosta *et al*., 2022; Behairy *et al*., 2020; Rahimi Anbarkeh *et al*., 2019). Antioxidants support spermatogenesis and reduce testicular damage caused by oxidative stress (Behairy *et al*., 2020). The use of fungicides in agriculture is increasing. Since exposure to fungicides may cause infertility and inexpensive compounds such as the ones containing minerals and vitamins are likely to reduce the harmful effects of fungicides, this study looked into the effects of fungicide penconazole on the testes of rats and the protective effects of selenium and vitamin C against testicular injury. This is the first study to analyze this subject in Iran.

## MATERIALS AND METHODS

### Animals and study design

This article was extracted from a research project given certificate approval no. IR.IAU.BEHBAHAN.REC.1400.007 by the Ethics Committee of the Islamic Azad University at Behbahan. In this experimental study, 54 male Sprague-Dawley rats were divided into nine groups of six subjects each. The study was performed in accordance with the standards stipulated for the use of laboratory animals and was approved by the Ethics Committee of the Islamic Republic of Iran. The rats were kept in polycarbonate cages on a cycle of 12 hours of light and 12 hours of darkness at a room temperature of 25°C; they had easy access to standard chow and water, and were kept in these conditions for ten days prior to the start of the study to adapt to the environment. To determine the lethal dose of penconazole, injections with 100 and 150 mg/kg of commercially available penconazole (Merck, Darmstadt, Germany) were administered to two groups of four rats. The rats given a dose of 100 mg/kg of penconazole developed muscle weakness and shortness of breath; half of the rats given 150 mg/kg of penconazole died. The lethal dose for male rats was thus set at 150 mg/kg. Therefore, the doses administered in this study were below 100 mg/kg. The rats were divided into nine groups, as follows: control; Se 0.5 mg/Kg; Vit C 100 mg/Kg; Penconazole 50 mg/kg; Pen 75 mg/Kg; Pen 50 mg/Kg +Se; Pen 75 mg/Kg +Se; Pen 50 mg/Kg + Vit C; Pen 75 mg/Kg +Vit C. Blood and tissue samples were collected. All rats were provided equal care and administered intraperitoneal injections for 14 days, with the exception of the control group. The injections were given with insulin syringes to cause the least pain to the animals. Anesthetics were not used. At the end of the injection period, the rats were placed on a 28-day rest period. This period is required so that a half-cycle of spermatogenesis, two cycles of sperm-forming epithelium, and two cycles of germ cells passing through the epididymis may occur. At the end of the rest period, the rats were weighed and killed with chloroform. Blood samples were taken from the left ventricle from the upper part of the abdominal cavity using a 5-ml syringe. The samples were then centrifuged for 15 min and the serum was kept at -20°C until use.

### Histopathology

The lower abdomen of the rats was opened under sterile conditions and the right testicle was removed. The testis was weighed using a digital scale and placed in a Bouin fixation solution for 18 hours for histological analysis. After fixation, 5-µm slices of paraffin-embedded tissue specimens were cut using a microtome and stained with hematoxylin-eosin (H&E). Then the diameter of the spermatogenic epithelium of specimens from experimental and control groups was measured using a light microscope. In order to count the number of sperm, one end of the right epididymis was cut immediately with sterile scissors after removal from the body and placed in 4 ml of normal saline buffer as an isotonic solution and incubated for 5 minutes at 37°C. This is the time it takes for the sperm to leave the duct completely. A drop (15µl) of the sperm suspension obtained in the previous step was placed on a hemocytometer slide and the number of sperms was counted.

### Biochemical Assays

Serum levels of LH and FSH were measured with ELISA kits (2100-Stat Fax, USA) on an ELISA reader (Finetest, China). The LH kit had a sensitivity of more than 0.938mU/ml and a range of 1.563-100/mU/ml; the FSH kit had a sensitivity of less than 1.406mU/ml and a range of 1.344-150mU/ml.

### Real-time PCR

β-actin, GPX4, AQP7, PRM2, and BAX gene expression was evaluated using real-time PCR in the left testis. The left testicle was removed through a slit in the lower abdomen and divided into four parts with a scalpel soaked in absolute alcohol. The specimens were placed on a petri dish and immediately frozen at -70°C. RNA was extracted using a commercial kit (Parstous Company, Iran). RNA quality was analyzed with agarose gel electrophoresis. cDNA was synthesized using a cDNA synthesis kit (Parstous Company, Iran). Quantitative real-time PCR tests were carried out on a Rotor-Gene 6000 system (Corbett Life Science, Mortlake, Australia) in a total volume of 10 µl including: 20 ngµl -1 of cDNA product, 10 pmol/µl -1 of each primer (β-actin: ACATCTGCTGGAAGGTGGAC, TTGCTGACAGGATGCAGAAG; GPX4: CACCACGCAGCCCTTCTTAT, GGCAGGAGCCAGGAAGTAATC; AQP7: AGGTCACGGGATGGGTTGATTG, ATGGTGTTTGGCCTTGGTTCCG; PRM2: TTGGCTCCAGGCAGAATG, ACAAGAGGCGTCGGTCAT and BAX: CGGAGGAAGTCCAGTGTCC, CCAGGATCGAGCAGAGAGG), 5 ml of SYBR premix ExTaq II (TaKaRa, Kusatsu, Shiga Prefecture, Japan). Real time PCR was carried out with the following protocol: 95°C for 4 min; 35 cycles at 94°C for 30s; 57°C for 30s; and 72°C for 30s.

### Statistical analysis

Data were analyzed using SPSS version 20 (Chicago, IL, USA) and ANOVA and T-tests. Real-time PCR data were analyzed using the ^∆∆CT^ method, where CT is the cycle threshold. For all the tests, *p*≤0.05 indicated statistical significance.

## RESULTS

The relative weight of the testicles and percent body weight change in the groups receiving penconazole separately were lower than the other groups; these variables increased with the addition of selenium and vitamin C, but there was no significant difference compared to the control group (*p*>0.05) (Table 1).

**Table 1:** The effect of treatment with selenium and vitamin C and/or penconazole on body weight and relative testicular weight of rats.

Groups	Body weight comparison (Mean±SD)	Relative testicular weight (Mean±SD)
**Control**	14.4±1.3	1.12±0.02
**Se 0.5 mg/Kg**	15.6±3.2	1.15±0.03
**Vit C 100 mg/Kg**	15.8±1.16	1.17±0.01
**Pen 50 mg/Kg**	13.5±2.1	0.98±0.1
**Pen 75 mg/Kg**	8.89±3.42	1±0.05
**Pen 50 mg/Kg +Se**	20.6±1.53	1.08±0.02
**Pen 75 mg/Kg +Se**	16.6 2.35	1.01±0.12
**Pen 50 mg/Kg +Vit C**	21.9±2.36	1.11±0.02
**Pen 75 mg/Kg +Vit C**	18.1±5.67	1.05±0.15

There was no significant difference between the diameter of the spermatogenic epithelium in groups receiving selenium and vitamin C and the control group (*p*>0.05), but in all experimental groups, a significant decrease in seminiferous epithelium diameter was observed (*p*<0.001). There was no significant difference in the comparison of seminiferous tubule diameter in the selenium and vitamin C groups compared to controls (*p*>0.05), but the experimental groups saw a significant increase in seminiferous tubule diameter compared to controls (*p*<0.05) (Table 2).

**Table 2:** The effect of treatment with selenium and vitamin C and/or penconazole on the diameter of seminiferous tubules and seminiferous epithelium.

Groups	Diameter of seminiferous epithelium (Mean±SD)	Diameter of seminiferous tubules (Mean±SD)
**Control**	91.2±2.2	280.3±8.4
**Se 0.5 mg/Kg**	94±3.6	274.3±4.1
**Vit C 100 mg/Kg**	95±2.8	267.3±2
**Pen 50 mg/Kg**	42.1±2.1	376.4±4.6
**Pen 75 mg/Kg**	40±2.8	390.3±3
**Pen 50 mg/Kg +Se**	48.3±2.9	321.4±3
**Pen 75 mg/Kg +Se**	45.6±2.9	356.5±4.8
**Pen 50 mg/Kg +Vit C**	48.5±1.9	286.3±4.3
**Pen 75 mg/Kg +Vit C**	47.3±3	293.2±2

The number of spermatogonia and primary spermatocytes in the groups receiving penconazole and penconazole with selenium or vitamin C were significantly lower than in the control group (*p*<0.05). The number of Sertoli cells in groups receiving selenium and vitamin C were similar to that of controls, but the number of Sertoli cells in groups receiving penconazole was lower than that of controls (*p*<0.001). In the present study, the number of sperms ejected from the last 1 cm of the epididymis was significantly decreased in experimental groups (*p*<0.05) (Table 3).

**Table 3:** The effect of treatment with selenium and vitamin C and/or penconazole on the Epidydimal Sperm, Sertoli cell, Primary spermatocyte and Spermatogonia cell count.

Groups	Spermatogonia cell count (Mean±SD)	Primary spermatocytes (Mean±SD)	Sertoli cell count (Mean±SD)	Epidydimal Sperm count (Mean±SD)
**Control**	73.2±2.2	81.2±2.2	15±1.22	11845±825
**Se 0.5 mg/Kg**	73.7±4	88.3±4.7	15.5±0.666	12175±145
**Vit C 100 mg/Kg**	75.2±3.7	90.2±7	16.5±0.614	12485±155
**Pen 50 mg/Kg**	58b±3.1	69.2a±2.8	10.9^c^±0.333	5620^a^±345
**Pen 75 mg/Kg**	57.5b±1.4	68.8^a^±1	10^a^±0.73	5385^a^±55
**Pen 50 mg/Kg +Se**	59.7b±3.2	72.1^b^±2.8	11.2^a^±0.401	6675^b^±355
**Pen 75 mg/Kg +Se**	58.1a±3	71.5^b^±1	11^a^±0.614	6490^a^±370
**Pen 50 mg/Kg +Vit C**	69.2±2.6	78±4.7	13.8^c^±0.6	7430^b^±160
**Pen 75 mg/Kg +Vit C**	59.5±2.9	73.1^b^±3.6	13.2^c^±0.666	6785^b^±145

a= It shows a significant difference of *p*<0.001.

b= It shows a significant difference of *p*<0.01.

c= It shows a significant difference of *p*<0.05.

Analysis of the level of sex hormones showed that the levels of LH, FSH, and testosterone were lower in the groups receiving penconazole (50 and 75 mg/kg), although not significantly (*p*>0.05) (Table 4).

**Table 4:** The effect of treatment with selenium and vitamin C and/or penconazole on the level of serum sex hormone.

Groups	LH (Mean±SD)	FSH (Mean±SD)	Testosterone (Mean±SD)
**Control**	4.64±0.69	2.39±0.07	0.88±0.09
**Se 0.5 mg/Kg**	5.06±0.58	2.45±0.05	0.89±0.03
**Vit C 100 mg/Kg**	5.10±0.48	2.49±0.17	0.96±0.07
**Pen 50 mg/Kg**	3.79±0.03	2.32±0.04	0.68±0.10
**Pen 75 mg/Kg**	3.70±0.12	2.17±002	0.57±0.02
**Pen 50 mg/Kg +Se**	4.34±1	2.32±0.04	0.83±0.07
**Pen 75 mg/Kg +Se**	3.94±0.36	2.32±0.12	0.71±0.06
**Pen 50 mg/Kg +Vit C**	4.64±0.51	2.39±0.07	0.85±0.04
**Pen 75 mg/Kg +Vit C**	4.55±0.06	2.36±0.04	0.81±0.18

### Gene expression

**Figure 1:**
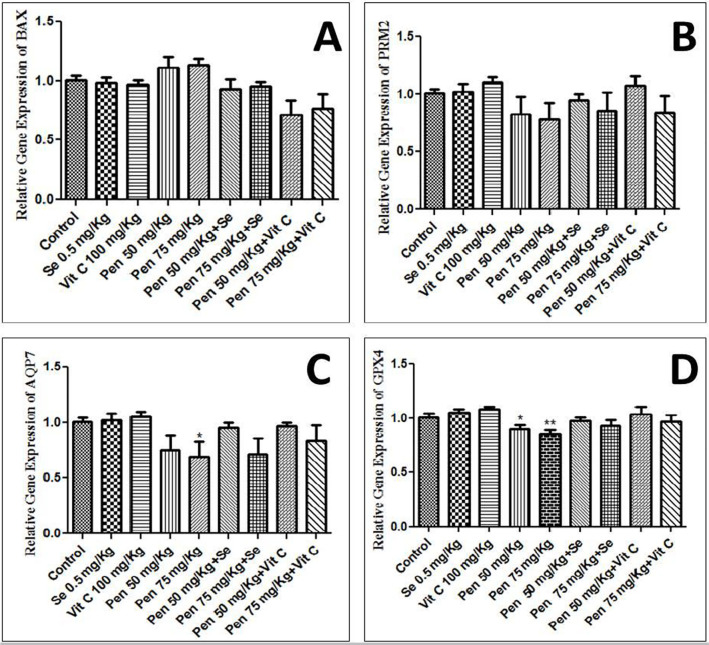
GPX4, AQP7, PRM2 and BAX gene expression in groups receiving selenium, penconazole and vitamin C compared with controls.

GPX4, AQP7, PRM2 and BAX gene expression analysis did not show significant changes in gene expression in most groups in comparison to controls. In the groups given 50 and 75mg/kg penconazole, GPX4, AQP7, PRM2 expression was decreased and BAX expression was increased when compared to the control group (Figure 1).

## DISCUSSION

According to the World Health Organization (WHO), around three million cases of poisoning and up to 220,000 deaths occur annually in connection with pesticides (Ghimire *et al*., 2022; Wang *et al*., 2019). Furthermore, published studies have reported nephrotoxicity, neurotoxicity, hepatotoxicity, carcinogenicity, mutagenicity, and endocrine disrupting effects in cases of exposure to pesticides (Georgiadis *et al*., 2018; Richardson *et al*., 2019; Alarcan *et al*., 2020; Martyniuk *et al*., 2020). Chemicals including drugs, pesticides, insecticides and different kinds of antibiotics with extensive presence in the environment are endocrine disruptors (Lauretta *et al*., 2019). In recent years, the use of antioxidant compounds is increasing rapidly and natural compounds are considered the most important reservoirs of antioxidants (Gulcin, 2020). Jamali *et al*. (2018) found that vitamin C decreases histological injury caused by methotrexate, and Abdel-Latif *et al*. revealed that vitamin C, and have antioxidant effects on oxidative damage associated with use of cisplatin (Abdel-Latif *et al*., 2022). Our study investigated the protective effect of selenium and vitamin C against the testicular damages caused by triazole fungicide penconazole. El-Sharkawy & El-Nisr (2013) found that penconazole at concentrations of 100 mg/kg significantly reduced the testosterone levels in mice. In another study, Lundgaard Riis *et al*. (2022) reported that conazoles have no effect on serum hormones levels including FSH, LH, and testosterone.

In this study, penconazole in concentrations of 50 and 75 mg/kg decreased the level of sex hormones in adult male rats; administration of selenium and vitamin D compensated for this reduction. Borai *et al*. (2019) found that penconazole significantly reduced the level of LH and FHS and increased the level of testosterone in male rats compared to controls; as seen in our study, the authors found that sesame seed oil normalized hormone levels. Marin-Guzman *et al*. showed that selenium deficiency in rats decreased the number of spermatozoa inside the seminiferous tubules and impaired sperm motility (Qazi *et al*., 2019). Atig *et al*. (2017) reported that selenium and vitamin E played an important role in improving sperm quality and motility in men. In the present study, the groups receiving penconazole 50 and 75 mg/kg had the lowest relative testis weight; diameter of spermatogenic tubules and seminiferous epithelium; number of epididymal sperm, Sertoli cells, primary spermatocytes cells, and spermatogonia cells. The rats given penconazole along with selenium and vitamin C had these negative effects compensated. The results of real time PCR did not show significant changes in GPX4, AQP7, PRM2 and BAX expression but, in the groups receiving penconazole 50 and 75 mg /kg, GPX4, AQP7, PRM2 expression was decreased and BAX expression was increased, thus supporting the existence of a toxicity effect of penconazole. Gene expression was modulated in the groups given penconazole along with selenium and vitamin C.
